# Promoting Intern Resilience: Individual Chief Wellness Check-ins

**DOI:** 10.15766/mep_2374-8265.10848

**Published:** 2019-10-25

**Authors:** Jason Fischer, Aviva Alpert, Priyanka Rao

**Affiliations:** 1 Pediatric Emergency Medicine Fellow, Division of Emergency Medicine, Children's Hospital of Philadelphia; 2 General Pediatrician, Children's Community Pediatrics Allegheny, University of Pittsburgh Medical Center; 3 Clinical Lecturer, Department of Pediatrics, Michigan Medicine

**Keywords:** Residency, Training, Pediatrics, Wellness, Intern, Resilience, Chief Resident, Burnout, Near-Peer Education, Communication Skills, Well-being/Mental Health

## Abstract

**Introduction:**

Promoting resilience is key during intern year as residents transition to becoming clinical providers. Residents consistently demonstrate a decline in empathy and an increase in burnout throughout training. Interventions involving mindfulness, stress management, and small-group discussions can reduce burnout. We created a curriculum to normalize the intern experience and provide debriefing opportunities to further improve resilience and decrease burnout.

**Methods:**

Thirty-two interns met monthly, one-on-one, with a pediatric chief resident to discuss personal, professional, and emotional well-being and complete just-in-time resilience exercises. After 6 and 12 months, we conducted follow-up surveys containing 5-point Likert questions and open-ended questions to determine interns' perceptions of the initiative.

**Results:**

We obtained response rates of 44% (14 interns) and 38% (12 interns) for the 6- and 12-month surveys, respectively. Interns found the sessions helpful for normalizing the intern experience (6 months: 4.6 ± 0.7, 12 months: 4.8 ± 0.5), stress management (6 months: 4.0 ± 1.0, 12 months: 4.3 ± 0.7), and feeling connected to program leadership (6 months: 4.6 ± 0.9, 12 months: 5.0 ± 0.0). Thematic analysis identified normalizing the intern experience, ability to express concerns, and mentorship as benefits.

**Discussion:**

Normalization of the intern experience and targeted wellness and resilience exercises can have a positive impact on interns' satisfaction with program support for their well-being. Through a time-limited intervention, chief residents can be utilized in a mentorship role that is well received by interns and rewarding for the chief residents.

## Educational Objectives

By the end of this activity, learners will be able to:

1. Normalize the intern experience and debrief the difficulties of intern year.
2. Develop a peer mentorship relationship with a chief resident.
3. Promote resilience and wellness via designated curricular resilience-building activity.

## Introduction

The body of literature on physician health and wellness has grown exponentially over the past decade. Recognizing the importance of this topic, the American Academy of Pediatrics (AAP) released a clinical report in 2014 that acknowledged the high rate of burnout among pediatric practitioners and trainees and called for pediatricians to be part of the national movement promoting physician well-being.^1^ The AAP and other national medical organizations have also recognized and emphasized that humanistic values and empathy are essential aspects of graduate medical training.^2,3^ Other specialties have also shown that patients of humanistic clinicians have better outcomes, increased satisfaction, and better adherence to treatments.^4,5^ However, the current climate reflects a well-documented decline in empathy among residents and increased burnout likely due to long work hours, high levels of stress, sleep deprivation, and lack of time for social activities outside of the hospital.^2,6–8^ Prior studies have demonstrated that resident burnout negatively impacts perceived quality of patient care,^9,10^ professionalism,^11^ and self-reported rates of medical errors.^12^ Looking specifically at pediatric residents, Baer et al.^8^ found that almost two in five pediatric residents experienced burnout and that those with burnout had higher odds of providing lower-quality care. For example, residents who were identified as having burnout were seven times more likely to make treatment or medication errors not related to inadequate knowledge, six times more likely to feel guilty about how they treated a patient, four times more likely to report having little emotional reaction to a patient's death, and four times more likely to discharge a patient early to make an inpatient service more manageable.^8^ This study also demonstrated that residents with burnout were nine times more likely to pay “little attention to the social or personal impact of an illness on a patient.”^8^ In similar studies that looked at first-year residents across multiple specialties, the rates of meeting criteria for clinical depression rose from 4% to 26%.^13^ Once burnout and depression are present, they can persist throughout residency and beyond.^14,15^

Seeing this trend nationally, within ourselves, and within our residency program, we examined interventions with proven efficacy to reduce burnout. We recognized that well-being, mental health, and empathy must be addressed during residency training to promote healthier trainees who provide optimal care to themselves and patients. Interventions previously shown to be effective involve mindfulness, stress management, and small-group discussions.^16–18^ Similarly, near-peer mentoring has been proposed as a way to decrease burnout among residents; however, this has not been studied on a larger scale.^19^ Although numerous frameworks for resilience exist, we chose to utilize Rosenberg's professional resilience theory,^20^ which posits that resilience is “the process of harnessing the resources we need to sustain well-being.” These include external, internal, and existential resources.^20^ Using this framework, we created a novel curriculum for first-year pediatric residents to normalize the intern experience and provide debriefing opportunities to improve their access to all three types of resilience resources and, in turn, decrease burnout.

The curriculum consisted of monthly one-on one meetings between a chief resident and a pediatric intern. Each meeting's purpose was threefold: (1) to check in on how the specific monthly rotation was going (providing a source of external support), (2) to inquire about the intern's mental health and self-care (to promote accessing both external and internal resources), and (3) to build resilience through predesigned activities (increasing access to different resources based on each specific activity). Although prior *MedEdPORTAL* publications have included curricula to foster resident resilience, these have focused on less frequent (three to four times per year) and more small-group (10-12 residents) interventions.^21,22^ Our intervention adds to the body of work on building resilience and decreasing burnout by focusing on a novel peer-mentoring model that incorporates skill-building exercises drawn from the existing literature on resilience and positive psychology. By pairing chief residents with individual interns, this model allows for a truly individualized experience while creating a sense of belonging and personal connection to administration for the trainee. This intervention was incorporated along with the already existing wellness curriculum in place for all residents in our program. The broader wellness curriculum involved bimonthly conferences, half of which were scheduled as times for residents to meet with one of the pediatric psychiatry attendings, who would facilitate discussions on burnout, managing challenging patient cases, and dealing with patient death, among other topics. The other half of the sessions were focused on more didactic topics including an introduction to mindfulness, creative activities such as painting International Classification of Diseases codes, healthy cooking/eating, and yoga class.

## Methods

We randomly assigned all first-year pediatric and medicine-pediatric interns (*n* = 32) to a pediatric chief resident mentor (*n* = 3) during orientation prior to the start of intern year. This equated to each chief resident mentoring 10-11 residents. The spring prior to the program, the pediatric chief residents attended the Accreditation Council for Graduate Medical Education–sponsored chief resident preparation course, a 3-day retreat designed to build the necessary skills to manage interpersonal dynamics commonly encountered as a chief resident. In June, during intern orientation, all interns attended a wellness session led by the pediatric chief residents and pediatric program director that included four activities (writing a letter to themselves, brainstorming exercise on what it means to be a doctor using a word cloud, circle of support activity, and introduction to mindfulness activity; Appendix A). Beginning in July, categorical pediatric interns and pediatric neurology interns met monthly with their chief mentor, whereas medicine-pediatric interns met for the 6 months of the year when they were assigned to pediatric rotations. Each meeting lasted 20-40 minutes. Meetings were scheduled via a Doodle poll and occurred in a location convenient to the intern. Often, the chief resident would meet the intern in the team room where the resident was working and then go with the intern to a more private meeting location (ideally with a window overlooking the arboretum to further promote wellness). If an intern did not sign up via the Doodle poll, the chief resident would contact the intern via a gentle personal email to remind the intern to sign up for a meeting. In addition, a previously determined resilience and wellness topic was discussed at each meeting. Three chief residents created this curriculum with additional support and insight from the pediatric program director, associate program directors, and a pediatric psychiatrist with expertise in physician burnout and resilience. We targeted different points during the year to provide just-in-time wellness/resilience topics pertinent to issues that interns would experience at that time of year (Appendix A). The activities attempted to help interns marshal internal, external, and existential resources to improve resilience. As with many mentorship programs, we started the year with introductory activities designed to allow the chief-intern pair to get to know each other on a more personal level and establish trust. Over the remainder of the academic year, we addressed different resilience and wellness topics, including self-care outside of work, perspectives on what it means to be a physician, ethical conundrums and tools to process such encounters, reflections on stress management, exercises in gratitude, recognition of signs and symptoms of burnout and strategies to mitigate it, coping with emotionally challenging patient experiences, and learning from medical mistakes. At the midpoint and end of the year, we focused the wellness and resilience topics on reflection on and evaluation of growth and development over the course of the year. We employed a variety of articles and activities to help emphasize key points of resilience and wellness (Appendix A).

To evaluate this intervention, we surveyed the interns on their perceptions of the program. We structured the survey using a combination of 5-point Likert scale questions and qualitative questions about the one-on-one chief meetings. We used Qualtrics to distribute the survey (Appendix B). The three chief residents designed the survey with input from the program director and associate program directors. We did not pilot the survey prior to implementation. Residents in all 4 years of our program received the survey to help provide an overall assessment of resident wellness, but only interns received the questions specifically targeting the intervention (questions 9-14). We designed the survey to assess interns’ perceptions of the initiative's impact on their resilience and ability to process the experiences of their intern year. To preserve the anonymity of the interns, we did not collect any individually identifiable information on the survey. We emailed an anonymous link to the survey to the intern listserve and subsequently sent one reminder email to complete the survey. The survey was open for 1 month for completion. We analyzed the Likert questions from the survey using Excel (version 16.13; Microsoft, Seattle, Washington). One author (Jason Fischer) reviewed the free-response questions.

## Results

All 32 pediatric and medicine-pediatric interns were mentored in the fashion described above. Approximately 90% of interns participated in all of the monthly meetings, and no individual missed more than one to two meetings over the course of the year. Forty-four percent (14 of 32) of the interns completed the 6-month survey, and 38% (12 of 32) completed the 12-month survey. Considering that responses were anonymous, no data were available to compare responders to nonresponders. Interns found the sessions helpful in all domains queried. In particular, interns found the sessions helped normalize the intern experience (4.6 ± 0.7 at 6 months and 4.8 ± 0.5 at 12 months), aided them in stress management (4.0 ± 1.0 at 6 months and 4.3 ± 0.7 at 12 months), and helped them feel connected to program leadership (4.6 ± 0.9 at 6 months and 5.0 ± 0.0 at 12 months; Table 1). More than 80% of responses at 6 months and 85% at 12 months agreed or strongly agreed with the statements that the meetings were helpful in the individual domains. The wellness and resilience-building topics were less well received compared to the other benefits of the intervention both quantitatively (3.9 ± 0.8 at 6 months and 3.6 ± 0.9 at 12 months) and qualitatively.

**Table 1.** Likert Rating^a^ for Chief-Intern One-on-One Sessions

Question	Midyear	End of Year
The sessions were helpful in:		
Normalizing the intern experience		
*M*	4.6 ± 0.7	4.8 ± 0.5
Agree/strongly agree (%)	93	100
Stress management		
*M*	4.0 ± 1.0	4.3 ± 0.7
Agree/strongly agree (%)	71	92
Providing me with additional wellness resources		
*M*	3.8 ± 1.1	4.1 ± 0.9
Agree/strongly agree (%)	64	67
Feeling connected to program leadership		
*M*	4.6 ± 0.9	5.0 ± 0.0
Agree/strongly agree (%)	93	100
Topics gave me tools to process intern year		
*M*	3.9 ± 0.8	3.6 ± 0.9
Agree/strongly agree (%)	79	67

a Rated on a 5-point Likert scale (1 = *strongly disagree*, 2 = *disagree*, 3 = *neutral*, 4 = *agree*, 5 = *strongly agree*).

Interns reported similar sentiments on the midyear and end-of-the-year surveys when asked about the sessions, with the majority of respondents providing responses to the open-ended questions (Table 2). Echoing the results of the Likert ratings, the meetings were well received. One intern wrote that it was a “favorite part of intern year.” Another wrote, “Fun and nice way to have some normalcy in this crazy year.” Multiple different themes emerged from the qualitative questions pertaining to debriefing and normalizing the experience, having a safe space to express concerns and feel supported, and the benefits of mentorship on intern year (Table 2).

**Table 2.** Qualitative Analysis of Open-ended Questions

	Respondents Who Replied	
	6-Month	12-Month
Question and Thematic Elements	Survey	Survey
What are your reflections on the one-on-one meetings?		
Overall	N/A	12/12 (100%)
Normalization of intern year	N/A	2/12
Connection to program leadership	N/A	4/12
Source of emotional support	N/A	5/12
Mechanism to provide program feedback	N/A	5/12
Tools to process intern year	N/A	3/12
What about these sessions has been helpful?		
Overall	12/14 (85.6%)	12/12 (100%)
Normalization of intern year	6/12	2/12
Connection to program leadership	1/12	1/12
Source of emotional support	6/12	9/12
Mechanism to provide program feedback	5/12	5/12
Tools to process intern year	6/14	5/12
How could the sessions be improved?		
Overall	13/14 (92.8%)	9/12 (75%)
Less structure	1/13	2/9
Less frequent	1/13	0/9
Supervisor pager/phone coverage	0/13	2/9
More feedback/advice	2/13	0/9
No suggestions	6/13	2/9
Which discussion topics have been helpful?		
Which were not helpful? What other topics would you want to see discussed in these meetings?		
Overall	4/14 (28.6%)	10/12 (83.3%)
Humanizing the experience	1/4	0/10
Being reminded about self-care	0/4	1/10
Stress management	1/4	1/10
Discussing patient deaths	0/4	0/10
Preparing to supervise	0/4	1/10
Mental health	0/4	2/10
Work/life balance	0/4	3/10
These topics were not helpful	0/4	2/10

Interns wrote that having a safe space and dedicated time to debrief were vital to their intern experience. One intern commented on this about the sessions: “[They] forced me to reflect on each month and discuss any problems. I would not have done this otherwise.” Another said the sessions were “a dedicated time to escape work and reflect on how the year was going.”

Many commented that the sessions helped normalize the intern experience and validated that others experienced similar sentiments. One intern reported that it was helpful to “understand that all interns go through this.” Another said, “Being able to normalize the experience with an older mentor enabled me to see the light at the end of the tunnel.” In an end-of-the-year reflection, one intern wrote,

The meetings made a huge impact this year. They helped me feel supported and well connected with administration. I felt comfortable asking for help or advice and informing the chiefs when things were not working well because of these meetings.

Having a safe space to express concerns, as well as feeling connected to the chiefs and, by extension, program leadership, was another benefit identified by many. One intern wrote, “The regular, one on one, set up of the meetings makes the intern feel connected and well supported by the residency program.” Another wrote that the meetings helped by “knowing that someone in leadership ‘has your back.’”

Finally, many commented on the role the chiefs played as mentors. One intern wrote that “being able to ask questions and express my concerns about how this year is going” was very helpful. Another expressed about the meetings that it felt “like there is somebody else who understands my situation and is able to give constructive feedback having recently gone through it themselves.” “Feels good to have someone else understand intern year” was another comment. At the end of the year, one intern commented that the chief presence as a mentor “enabled me to ask questions and validate some of the things I was worried about as the year went on.” Another commented that it was helpful to have “some dedicated time to give/get feedback.”

The major criticism of the meetings came when interns were asked about the wellness topics:

I did not feel that the specific topics were the portion of the meetings that were helpful. The most helpful part of the meeting was the freedom to openly discuss how intern year was going and how I was feeling and to ask for advice on how to handle particularly difficult situations.

However, there were some topics interns found helpful. “I thought the discussion about patient deaths was a good conversation and helped me frame my own thoughts,” wrote one intern, whereas another liked “some of the early topics regarding life balance and how to split it up.”

Overall, the meetings were well received, and interns commented that they helped them process the intern experience and feel supported by the program, gave them an opportunity to check in on their own mental health so that it did not slip through the cracks, and provided them with a mentor to whom they could go for advice or help.

## Discussion

The first year of residency is a time of immense growth and distinct challenge for new trainees. Through a novel curriculum providing individualized evaluation and support, we helped residents process their experiences, develop new coping skills, and mitigate burnout throughout the year. Although there are many commonalities in the challenges faced by interns, there are also numerous personal factors that modulate an individual's experience. By focusing our intervention on one-on-one meetings, we could better meet the needs of the individual trainee by helping each marshal the appropriate external, internal, and existential resources that the individual needed. Furthermore, by having frequent scheduled check-in times, we improved early identification of individuals needing additional levels of support or intervention. Through both qualitative assessment and quantitative evaluations, we showed that the interns in our program perceived the monthly meetings to be valuable. Specifically, the meetings helped interns adjust to residency, provided normalization of experiences at different time points, connected residents to needed resources, and gave residents an increased sense of belonging.

We encountered several challenges during the implementation of the intervention. Given the busy and unpredictable daily schedule of the interns, it was sometimes hard for them to commit in advance to a time for the monthly meeting. We attempted to make scheduling as easy as possible by coming directly to each intern's work space in the hospital and by being flexible with last-minute changes in the meeting times. Occasionally, interns would resist meetings due to a busy clinical load. It was important to maintain a careful balance between respecting the intern's clinical responsibilities while also encouraging and sometimes requiring them to take time out of their day for the meeting. We learned that it was often those interns who resisted the meetings most who were actually in greatest need of mental health support. Overall, the interns almost universally reported that they found the meetings helpful and a good use of their time.

Additionally, our intervention was dependent on a time commitment from the three chief residents. Chief residents have many competing demands on their time, including clinical, educational, and administrative responsibilities. Although the overall time requirement of 4-6 hours per chief resident per month was felt to be manageable, it did require a dedicated commitment. Having support from the program administration and clinical faculty was extremely important, and the backing of our program director was particularly critical to the success of the intervention. Despite the time commitment, this was a highlight of the year for each of the chief residents.

This intervention also relied on cultivating one-on-one personal relationships between interns and chief residents. Although our group did not encounter conflict between interns and chief residents, we do understand that this reflects only our personal group of chiefs and intern relationships. When conflicts arose between faculty mentors and residents at our institution, the residency program administration evaluated each conflict on a case-by-case basis. Occasionally, the mentee was reassigned, but more often, the mentor received feedback and coaching from program administration on how to more effectively connect with that mentee. Although we did not encounter any conflicts, a plan for dealing with them, should they arise, would be a prudent addition to our program.

Because of these meetings, interns shared a significant amount of personal information with the chief residents. Prior to initiating the sessions, we did not develop a uniform policy for handling situations that might require a breach of confidentiality. Instead, similar to potential interpersonal conflicts, we left each situation to the discretion of the individual chief resident. When chiefs felt that information did need to be shared with program administration, we relied on our established relationships with individual interns to be honest and transparent about the reasons we needed to share that information, especially when there were concerns about the safety or mental health of the trainee. A more formal policy should be developed and shared with the interns in future iterations of this initiative.

Although the results from our follow-up evaluation clearly demonstrated perceived value in the intervention, we were not able to fully assess for a direct correlation between the intervention and levels of burnout throughout intern year. Further evaluation would be needed to determine if the program directly helped reduce burnout. The specific wellness activities done at each meeting were variably received. Some interns found these activities helpful, whereas others felt they were a less valuable use of time. Additional follow-up would be needed to determine which activities were most beneficial and to see how the curriculum could be further developed to meet the desired goals.

One major limitation of our survey is its low response rate. There are many possible reasons for this. Despite the anonymous nature of the surveys, there is an inherent power dynamic between chief residents and interns. Interns may fear reprisal from chief residents in charge of scheduling and other administrative tasks. Interns may also fear inadvertently identifying themselves with their comments, leading to a negative perception of them by the residency program and potentially impacting future employment. We attempted to mitigate this as much as possible with the 12-month surveys coming from outgoing chief residents who would not have further involvement in program administration. Additionally, the surveys were sent out after schedules and vacation requests for the coming year had already been processed. Despite these attempts to mitigate these fears, the low response rate could introduce bias into the results.

Further work is needed to continue developing interventions that successfully decrease burnout in trainees and provide the needed support for trainees to build and maintain resilience during medical training and throughout a medical career. Additional evaluation is needed to assess the benefits of this particular intervention in reducing rates of burnout during intern year and during future years of training. Future development of this curriculum could focus on how to modify the content of the meetings to better address both individual needs and common challenges of trainees.

Overall, the results of this intervention show that individual mentorship from chief residents can have an extremely positive impact on the intern experience. Moreover, these findings support the value of incorporating personalized mentorship and wellness curricula into medical training programs more broadly. Advances in educational policy that help support infrastructure for such programming could have positive implications for medical trainees across disciplines.

## Appendices

A. Session Instructions.docx
B. Wellness Curriculum Survey.docx All appendices are peer reviewed as integral parts of the Original Publication.
