# Supplementary material for: Promoting Intern Resilience: Individual Chief Wellness Check-ins
Source: MedEdPORTAL. 2019 Oct 25;15:10848. doi: 10.15766/mep_2374-8265.10848 (PMC6946579; doi:10.15766/mep_2374-8265.10848)
Supplement: Supplementary file 1 — A. Session Instructions.docx B. Wellness Curriculum Survey.docx [file mep-15-10848-s001.zip › A. Session Instructions.docx]

**Overview of Monthly Sessions:**

| **Month** | **Wellness Topic** | **Resources** |
| --- | --- | --- |
| June | Intern Orientation   - Interns write letter to themselves - Word cloud “What does it mean to be a doctor” - Circle of Support - Introduction to mindfulness | Handout: Circle of Support Template^1^ |
| July | Get to know you activity, intro to meetings | Article: “36 Questions to Fall in Love”^2^ |
| August | What is the one activity you do outside of work that you really enjoy and are making a priority this year? |  |
| September | What does it mean to you to be a doctor?  How has this changed since your intern year started? | Article: “To Isaiah”^3^ |
| October | Have you encountered any ethical conundrums in pediatrics? What has helped you work through them/think about them/reflect on them? |  |
| November | How do you deal with stress? Provide reflections on stress management |  |
| December | 1. Check-In: Self-Care Prioritization & Non-Work Activities.  2. Midway point Re-Do/Reevaluate “Circle of Support”  3. Give interns letters that they wrote to themselves | Handout: Circle of Support Template^1^ |
| January | For what in your life do you feel most grateful? |  |
| February | Have you thought about resident burnout? Do you know any of the signs and symptoms? Talk about sign/symptoms of burnout and strategies to help mitigate |  |
| March | What strategies do you employ when coping with emotionally challenging patient experiences? | Article: “Death and Sandwiches”^5^  Article: “How to tell a Mother her Child is dead”^6^ |
| April | What do you do when you make a mistake? | Article: “What I Learned About Adverse Events from Captain Sully”^7^ |
| May | Have the intern-chief meetings been helpful? Ways to improve for next year’s interns? Reflection on intern year and closure of intern-chief meetings. |  |

**Orientation Wellness Session:**

This session should be scheduled during intern orientation and last for ~45 minutes. It should be led by the program director (with inclusion of assistant/associate program directors as appropriate) and the chief residents. Below is an annotated outline of activities:

1. Welcome and Introduction – 5 minutes
   1. Program director welcomes all interns to the session
   2. Introduce the concept of wellness during residency
2. Exercise on “What Does it Mean to be a Doctor” – 10 minutes
   1. Ask interns to brainstorm five words that describe what it means to be a doctor to them and write them on individual pieces of paper
   2. Collect the papers and choose to either type them into a free online world cloud generator (<https://www.wordclouds.com/>) or as an alternative to the online generator facilitators may also tally words collected from residents by hand before discussing.
      1. When words are repeated they show up larger on the word cloud compared to words that are only entered once. Similarly if collected by hand you may place further emphasis on words that are written down multiple times.
   3. Display word cloud on the big screen and allow everyone time to look over all the different words that were chosen by their peers.
   4. Chief residents facilitate a large group discussion about the different meanings of doctor to different individuals
3. Circle of Support Activity^1^ – 10 minutes
   1. Draw a version of the circle of support on the white board at the front of the room and distribute paper copies of the template to each intern (see paper template sample below)
   2. Directions for activity:
      1. Consider yourself as the middle of the circle with layers of support rippling outwards
      2. Think about where you are currently and what people, places, material items, and ideas/beliefs are supporting you.
      3. Reflect on the upcoming intern year and how you think some of these supports may change over the course of the year and draw arrows reflecting if the support will move closer to your inner circle or further away on to your outer circle
      4. Remember that this is a completely personal activity and reflection and what supports you may not be the same as what supports your colleague
   3. Provide examples in each domain of how support may change over the course of intern year
   4. Ask interns to complete their own circle of support
4. Introduction to Mindfulness -- 10 minutes
   1. Introduce the concept of mindfulness by providing each resident with a chocolate covered cherry and asking them to focus on tastes as each layer of the chocolate and then cherry emerge
   2. Discuss how even small moments of mindfulness (drinking coffee in the morning, soothing activity on drive into work, taking a one-minute breathing break during a busy day) can help to refocus and energize you on busy days
5. Writing a letter to themselves – 5 minutes
   1. Distribute a card and envelope to each intern and ask them to write a letter to themselves. Inform them prior to writing the letter that they will receive them midway through intern year.
      1. Letters should reflect on thoughts and ideas they have as they start their intern year as well as anything else they’d like to include
   2. Interns address and seal their own letters to ensure privacy and then the letters are collected and stored until the 6 months into their year when they will be mailed to the interns.
6. Wrap up and time for questions – 5 minutes
   1. Wrap up and conclude activity by asking interns if they have questions or concerns about the wellness program
   2. Inform them that this program is designed with their wellness in mind so if they have suggestions as the year goes along for additional activities to include to please suggest them to leadership.

My Circle of Support


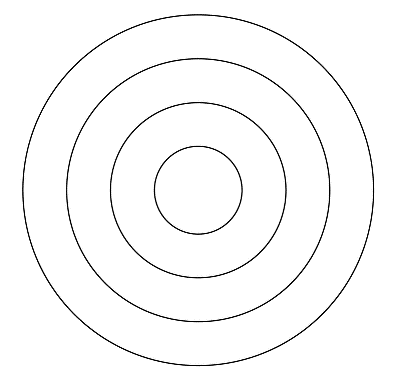


**July Chief Check-In Session:**

This session is the first of the individual check-ins between interns and their assigned chief resident. Interns are asked to sign up for a 40 minute time slot with their chief resident via a doodle poll. The chief residents should arrange a location convenient for the intern (recommended to meet them in their team room and walk to a quiet place with a window overlooking the outside of the hospital, preferably a green space). Below is an annotated outline of the first session:

1. Welcome and introduction to meetings – 5 minutes
   1. Chief resident re-introduces concept of the individual meetings to the intern and re-enforces that the meetings are designed with the intern’s wellness in mind, inviting them to share in making the sessions useful for them.
   2. Overview of typical session
2. Get-To-Know-You Activities – 5-10 minutes
   1. Chief Residents select 2-3 questions from the New York Times article, “36 Questions that Lead To Love”^2^ to use as an ice breaker activity. The chiefs also give the interns their answers so that the intern gets to know their chief resident on a personal level as well. Examples of questions:
      1. What would constitute a “perfect” day for you?
      2. For what in your life do you feel most grateful?
      3. If you could change anything about the way you were raised, what would it be?
      4. Your house, containing everything you own, catches fire. After saving your loved ones and pets, you have time to safely make a final dash to save any one item. What would it be? Why?
3. Rotation Check-In – 10-15 minutes
   1. Pediatric Chief Resident asks the intern how their current rotation is going and inquires about any issues both interpersonally, or with provision of care. This is often a time where systems issues or team dynamics are discussed.
   2. Pediatric Chief Resident helps the intern to develop skills to navigate team dynamics if appropriate and follows up with other issues with relevant parties after the meeting
4. Mental Health and Coping Check-In – 10-15 minutes
   1. Chief resident checks in with intern regarding how they are doing emotionally. This is a time where the interns can process difficult patient interactions, peer relationships, or stress outside of work.
   2. During the first quarter particular attention should be paid to adjusting to life in a new city, if applicable, their new role and responsibilities, and their changing support system.

**August Chief Check-In Session:**

This session is the second of the individual check-ins between interns and their assigned chief resident. Interns should, again, be asked to sign up for a 40-minute time slot with their chief resident via a doodle poll. The chief residents should arrange for a location convenient for the intern (recommended to meet them in their team room and walk to a quiet place with a window overlooking the outside of the hospital, preferably a green space). Below is an annotated outline of the second session:

1. Optional Ice-Breaker – 5 minutes
   1. Chief Resident uses another question from the New York Times article, “36 Questions that Lead To Love”^2^ as an ice breaker activity. Interns and chief residents share their responses with each other
2. Rotation Check-In – 10-15 minutes
   1. Pediatric Chief Resident asks the intern how their current rotation is going, inquires about any issues both interpersonally, or with provision of care. This is often a time where systems issues or team dynamics are discussed
   2. Pediatric Chief Resident helps the intern to develop skills to navigate team dynamics if appropriate and follows up with other issues with relevant parties after the meeting
3. Mental Health and Coping Check-In – 10-15 minutes
   1. Chief resident checks in with intern regarding how they are doing emotionally. This is a time where the interns can process difficult patient interactions, peer relationships, or stress outside of work.
   2. During the first quarter particular attention should be paid to adjusting to life in a new city, if applicable, their new role and responsibilities, and their changing support system.
4. Wellness Discussion Topic – 10-15 minutes
   1. What is the one activity you do outside of work that you really enjoy and are making a priority this year?
      1. Discussion on how to prioritize the activities interns do outside of work, understanding that they will have much less free time compared with medical school
      2. Guide interns in generation of a plan to incorporate those activities into their new lifestyle as some of the strategies they used as medical students may no longer apply.

**September Chief Check-In Session:**

This session is the third of the individual check-ins between interns and their assigned chief resident. Interns should sign up for a 40-minute time slot via a doodle poll and chief residents should arrange for a convenient location for the intern with the same parameters as previous meetings. Below is an annotated outline of the third session:

1. Day Prior To Session
   1. Email article “To Isaiah”^3^ to the intern with instructions to read the article prior to the meeting.
2. Rotation Check-In – 10-15 minutes
   1. Pediatric Chief Resident asks the intern how their current rotation is going, inquires about any issues both interpersonally, or with provision of care. This is often a time where systems issues or team dynamics are discussed
   2. Pediatric Chief Resident helps the intern to develop skills to navigate team dynamics if appropriate and follows up with other issues with relevant parties after the meeting
3. Mental Health and Coping Check-In – 10-15 minutes
   1. Chief resident checks in with intern regarding how they are doing emotionally. This is a time where the interns can process difficult patient interactions peer relationships, or stress outside of work.
   2. During the first quarter particular attention should be paid to adjusting to life in a new city, if applicable, their new role and responsibilities, and their changing support system.
4. Wellness Discussion Topics – 10-15 minutes
   1. What does it mean to you to be a doctor?
   2. How has this changed since your intern year started?
   3. What about the article (“To Isaiah” ^3^) resonates with you?
      1. Discuss the intern’s experiences thus far during intern year and how it has changed/shaped what it means to be a doctor.
      2. Focus is on helping them to navigate the emotional challenges of treating patients at their most vulnerable as well as coping with their own insecurities surrounding all that they do not know but perceive that they are expected to know.

**October Chief Check-In Session:**

This session is the fourth of the individual check-ins between interns and their assigned chief resident. As before, interns sign up for meetings and chief residents meet them in a convenient location. Below is an annotated outline of the fourth session:

1. Rotation Check-In – 10-15 minutes
   1. Pediatric Chief Resident asks the intern how their current rotation is going, inquires about any issues both interpersonally, or with provision of care. This is often a time where systems issues or team dynamics are discussed
   2. Pediatric Chief Resident helps the intern to develop skills to navigate team dynamics, if appropriate, and follows up on other issues with relevant parties after the meeting
2. Mental Health and Coping Check-In – 10-15 minutes
   1. Chief resident checks in with intern regarding how they’re doing emotionally. This is a time where the interns can process difficult patient interactions.
   2. During the second quarter, particular attention should be paid to fatigue, self-care and the notion of the “imposter syndrome” in order to normalize the intern experience.
3. Wellness Discussion Topics – 10-15 minutes
   1. Have you encountered any ethical conundrums in pediatrics?
   2. What has helped you work through them/think about them/reflect on them?
      1. Discussion on any ethical conundrums they have experienced thus far
      2. Focus is on navigating the interns through the challenging emotions of these conundrums and helping them to process and grow from those experiences through a facilitated discussion.

**November Chief Check-In Session:**

This session is the fifth of the individual check-ins between interns and their assigned chief resident. As before, interns sign up for meetings and chief residents meet them in a convenient location. Below is an annotated outline of the fifth session:

1. Rotation Check-In – 10-15 minutes
   1. Pediatric Chief Resident asks the intern how their current rotation is going, inquires about any issues both interpersonally, or with provision of care. This is often a time where systems issues or team dynamics are discussed
   2. Pediatric Chief Resident helps the intern to develop skills to navigate team dynamics if appropriate and follows up with other issues with relevant parties after the meeting
2. Mental Health and Coping Check-In – 10-15 minutes
   1. Chief resident checks in with intern regarding how they’re doing emotionally. This is a time where the interns can process difficult patient interactions.
   2. During the second quarter, particular attention should be paid to fatigue, self-care and the notion of the “imposter syndrome” in order to normalize the intern experience.
3. Wellness Discussion Topics – 10-15 minutes
   1. How do you deal with stress?
   2. Provide reflections on stress management style
      1. Discussion on how the intern manages stress.
      2. Ask them to reflect on how they think it works.
      3. Focus in discussion should be on promoting healthy stress management techniques and helping them to develop new skills/strategies that align with their personalities.
      4. Normalization of the stressful nature of the intern experience should also be included, acknowledging that the session is not meant to eliminate stress, but rather to promote healthy coping strategies and recognize when help is needed.

**December Chief Check-In Session:**

This session is the sixth of the individual check-ins between interns and their assigned chief resident. As before, interns sign up for meetings and chief residents meet them in a convenient location. Below is an annotated outline of the sixth session:

1. Two weeks prior to meeting – remind Program Director to mail interns the letters they wrote to themselves during orientation
2. Day Prior To Meeting – Email interns the “Circle Of Support” template^1^
3. Rotation Check-In – 10-15 minutes
   1. Pediatric Chief Resident asks the intern how their current rotation is going, inquires about any issues both interpersonally, or with provision of care. This is often a time where systems issues or team dynamics are discussed
   2. Pediatric Chief Resident helps the intern to develop skills to navigate team dynamics if appropriate and follows up with other issues with relevant parties after the meeting
4. Mental Health and Coping Check-In – 10-15 minutes
   1. Chief resident checks in with intern regarding how they are doing emotionally. This is a time where the interns can process difficult patient interactions.
   2. During the second quarter, particular attention should be paid to fatigue, self-care, and the notion of the “imposter syndrome” in order to normalize the intern experience.
5. Wellness Discussion Topics – 10-15 minutes
   1. Revisiting the Circle of Support Activity
      1. Discuss their ever-changing circle of support
      2. Re-evaluation of which supports are helpful now that they have had some time to adjust to their new role/life
      3. Acknowledging that some people in their life who previously may have provided tremendous support may not be providing the support that they need during intern year and that this does not make them less important people in their lives.
      4. Also acknowledging that the type of support they need during residency may be different than the type of support they had previously needed and that this too is normal.
   2. Check-in on how they are doing prioritizing the one or two activities they enjoy that they wanted to maintain during residency.
      1. Evaluate whether they have been able to maintain them
      2. If they have not been able to maintain them, asking whether those activities are still important
         1. If so, how they can be more deliberate about incorporating them.
         2. If not, brainstorm new activities that they enjoy and use to decompress
   3. Ask them to reflect on what they wrote in their letters during orientation and whether the experience thus far was what they expected.
      1. Focus should be on helping them to normalize the intern experience
   4. Reflections on the first half of the year
      1. Try to re-direct residents to focus on how far they’ve come since starting to reinforce a sense of accomplishment as a resilience building tool

**January Chief Check-In Session:**

This session is the seventh of the individual check-ins between interns and their assigned chief resident. As before, interns sign up for meetings and chief residents meet them in a convenient location. Below is an annotated outline of the seventh session:

1. Holiday Check-In – 5 minutes
   1. Discuss how the intern’s holiday was.
      1. Pay special attention whether their time off was rejuvenating or exhausting as this might provide clues to their current mindset
2. Rotation Check-In – 10-15 minutes
   1. Pediatric Chief Resident asks the intern how their current rotation is going, inquires about any issues both interpersonally or with provision of care. This is often a time where systems issues or team dynamics are discussed
   2. Pediatric Chief Resident helps the intern to develop skills to navigate team dynamics if appropriate and follows up with other issues with relevant parties after the meeting
3. Mental Health and Coping Check-In – 10-15 minutes
   1. Chief resident checks in with intern regarding how they are doing emotionally. This is a time where the interns can process difficult patient interactions.
   2. During the third quarter, particular attention should be paid to recognizing early symptoms of burnout, depression and compassion fatigue. Additional attention should be paid to addressing the “imposter syndrome,” as when patient volume and seasonal affective disorder peak, symptoms become hyper-acute.
4. Wellness Discussion Topics – 5-10 minutes
   1. Discussing the things in life the intern is grateful for
      1. In the midst of the busy clinical environment, cold weather and difficult training process, the things we appreciate and that nourish us often fall by the wayside
      2. Discussing with the intern what things they’re grateful for and how to make these people/things/activities/concepts a more central part of their everyday lives in order to redirect their focus from the negativity that can plague their day-to-day.

**February Chief Check-In Session:**

This session is the eighth of the individual check-ins between interns and their assigned chief resident. As before, interns sign up for meetings and chief residents meet them in a convenient location. Below is an annotated outline of the eighth session:

1. Rotation Check-In – 10-15 minutes
   1. Pediatric Chief Resident asks the intern how their current rotation is going, inquires about any issues both interpersonally, or with provision of care. This is often a time where systems issues or team dynamics are discussed
   2. Pediatric Chief Resident helps the intern to develop skills to navigate team dynamics if appropriate and follows up with other issues with relevant parties after the meeting
2. Mental Health and Coping Check-In – 10-15 minutes
   1. Chief resident checks in with intern regarding how they are doing emotionally. This is a time where the interns can process difficult patient interactions.
   2. During the third quarter, particular attention should be paid to recognizing early symptoms of burnout, depression and compassion fatigue. Additional attention should be paid to addressing the “imposter syndrome,” as when patient volume and seasonal affective disorder peak, symptoms become hyper-acute.
3. Wellness Discussion Topics – 5-10 minutes
   1. Frank Discussion on Burnout
      1. Asking the intern if they have thought about resident burnout, checking if they know any of the signs and symptoms
      2. Discuss sign/symptoms of burnout
      3. Guide the discussion towards strategies to help mitigate burnout

**March Chief Check-In Session:**

This session is the ninth of the individual check-ins between interns and their assigned chief resident. As before, interns sign up for meetings and chief residents meet them in a convenient location. Below is an annotated outline of the ninth session:

1. Day Prior To Session
   1. Articles “Death and sandwiches”^5^ and “How to tell a mother her child is dead”^6^ are emailed to the intern with instructions to read the articles prior to the meeting.
2. Rotation Check-In – 10-15 minutes
   1. Pediatric Chief Resident asks the intern how their current rotation is going, inquires about any issues both interpersonally, or with provision of care. This is often a time where systems issues or team dynamics are discussed
   2. Pediatric Chief Resident helps the intern to develop skills to navigate team dynamics if appropriate and follows up with other issues with relevant parties after the meeting
3. Mental Health and Coping Check-In – 10-15 minutes
   1. Chief resident checks in with intern regarding how they are doing emotionally. This is a time where the interns can process difficult patient interactions.
   2. During the third quarter, particular attention should be paid to recognizing early symptoms of burnout, depression and compassion fatigue. Additional attention should be paid to addressing the “imposter syndrome,” as when patient volume and seasonal affective disorder peak, symptoms become hyper-acute.
4. Wellness Discussion Topics – 5-10 minutes
   1. Challenging Patient Experiences – Challenging Patients and Patient Deaths
      1. Utilizing the two articles emailed out to the interns, ask if they have had a patient pass away this year. If they have, use this time as a reflection space for them on how they have processed the emotional turmoil surrounding their first patient deaths
      2. If time (or if interns have not experienced a patient death yet), use the time to discuss challenging patient/family encounters as these often plague interns at this time of year. Help them work through those interactions that they are still struggling with.

**April Chief Check-In Session:**

This session is the tenth of the individual check-ins between interns and their assigned chief resident. As before, interns sign up for meetings and chief residents meet them in a convenient location. Below is an annotated outline of the tenth session:

1. Day Prior To Session
   1. Article “What I learned about adverse events from Captain Sully”^7^ is emailed to the intern with instructions to read the article prior to the meeting.
2. Rotation Check-In – 10-15 minutes
   1. Pediatric Chief Resident asks the intern how their current rotation is going, inquires about any issues both interpersonally, or with provision of care. This is often a time where systems issues or team dynamics are discussed
   2. Pediatric Chief Resident helps the intern to develop skills to navigate team dynamics if appropriate and follows up with other issues with relevant parties after the meeting
3. Mental Health and Coping Check-In – 10-15 minutes
   1. Chief resident checks in with intern regarding how they are doing emotionally. This is a time where the interns can process difficult patient interactions.
   2. During the fourth quarter, particular attention should be paid to acknowledging the anxieties surrounding transitioning from the intern to the senior role and processing this transition with them.
4. Wellness Discussion Topics – 5-10 minutes
   1. What do you do when you make a mistake?
      1. Utilizing the article emailed out to the interns, ask if they have had to disclose a mistake to a patient.
      2. Discuss this process as well as the expectations of the system.
      3. Reflect on the article and how the stressful environment that we work in rarely affords time for reflection or mental decompression despite how vitally important they are.
      4. Use Captain Sully as an example of someone who expertly navigated a crisis situation, but still suffered from significant mental health and post-traumatic issues related to the experience.
      5. Discuss how this experience may seem extreme, but the daily toll that taking care of sick children can similarly affect the individuals providing that care.

**May Chief Check-In Session:**

This session is the eleventh and final individual check-ins between interns and their assigned chief resident. As before, interns sign up for meetings and chief residents meet them in a convenient location. Below is an annotated outline of the eleventh session:

1. Rotation Check-In – 10-15 minutes
   1. Pediatric Chief Resident asks the intern how their current rotation is going, inquires about any issues both interpersonally, or with provision of care. This is often a time where systems issues or team dynamics are discussed
   2. Pediatric Chief Resident helps the intern to develop skills to navigate team dynamics if appropriate and follows up with other issues with relevant parties after the meeting
2. Mental Health and Coping Check-In – 10-15 minutes
   1. Chief resident checks in with intern regarding how they are doing emotionally. This is a time where the interns can process difficult patient interactions.
   2. During the fourth quarter, particular attention should be paid to acknowledging the anxieties surrounding transitioning from the intern to the senior role and processing this transition with them.
3. Wellness Discussion Topics – 5-10 minutes
   1. Reflections and Transitions
      1. Acknowledge that these meetings have been meaningful to you (if this is authentic) and that closings and transitions can be difficult.
      2. Provide the intern an opportunity to discuss their reflections and feelings about the end of these meetings as well as their transition process from an intern into a second year.
      3. Ask for their honest feedback on the meetings, discussing what was helpful as a means of both debriefing the year as well as for quality improvement for subsequent years.
      4. (If desired) Acknowledge that while this is the last meeting the chief is still available to them as a resource (especially important if the chief will be leaving the institution). This statement serves two purposes
         1. Validates the significance of the relationship the chief and intern have developed
         2. Acknowledges that the relationship was authentic and reinforces the mentorship element for the intern.

**References:**

1. Leffler, M. (2016, May). *Circle of Support*. Professional development activity presented at Pediatric Leadership Skills Training Program for Chief Residents, Jacksonville, Florida.
2. Jones D. The 36 Questions That Lead to Love. *New York Times*. January 9, 2015.
3. Berwick DM. To Isaiah. *JAMA*. 2012;307(24):2597-2599. doi:10.1001/jama.2012.6911.
4. Khullar D. Death and Sandwiches. *New York Times*. https://well.blogs.nytimes.com/2015/12/10/death-and-sandwiches/. Published December 10, 2015.
5. Rosenberg N. How to Tell a Mother Her Child Is Dead. *New York Times*. September 3, 2016.
6. Stiegler MP. What I Learned About Adverse Events From Captain Sully. *JAMA*. 2015;313(4):361. doi:10.1001/jama.2014.16025.
