# Supplementary material for: Promoting Intern Resilience: Individual Chief Wellness Check-ins
Source: MedEdPORTAL. 2019 Oct 25;15:10848. doi: 10.15766/mep_2374-8265.10848 (PMC6946579; doi:10.15766/mep_2374-8265.10848)
Supplement: Supplementary file 1 — A. Session Instructions.docx B. Wellness Curriculum Survey.docx [file mep-15-10848-s001.zip › B. Wellness Curriculum Survey.docx]

Intern One-On-One Meetings

Start of Block: Default Question Block

We are looking to better understand how the residency program supports residents and how to continue to improve the resources we provide.  Please take a few minutes to complete the following survey.  The survey is anonymous, so please give as much honest feedback as possible.

Q1 What year are you?

- HO-1 (1)
- HO-2 (2)
- HO-3/4 (3)

Q2 The residency program supports me in the following areas:

|  | Strongly disagree (1) | Somewhat disagree (2) | Neither agree nor disagree (3) | Somewhat agree (4) | Strongly agree (5) |
| --- | --- | --- | --- | --- | --- |
| Career development (1) |  |  |  |  |  |
| Stress management (2) |  |  |  |  |  |
| Mental health and wellness (3) |  |  |  |  |  |
| Physical well-being (4) |  |  |  |  |  |

Q3 How has the program made you feel supported thus far?

________________________________________________________________

Q4 How could the program have done a better job supporting you?

________________________________________________________________

Q5 Currently, who do you rely on during times of stress?  Select your top two choices.

- Friends outside of residency (1)
- Co-Residents (2)
- Spouse/Significant Other (3)
- Family (4)
- Residency Program leadership (5)
- Other (6)

Display This Question:

If Currently, who do you rely on during times of stress? Select your top two choices. = Other

Q6 Please specify

________________________________________________________________

Q7 What types of activities do you do to unwind and destress? Select your top two choices.

- Exercise (1)
- Spend time with friends/family (2)
- Cook (3)
- Read/Watch Television (4)
- Spend time outdoors (5)
- Other (6)

Display This Question:

If What types of activities do you do to unwind and destress? Select your top two choices. = Other

Q8 Please list your other activities:

________________________________________________________________

End of Block: Default Question Block

Start of Block: One On One Meeting Questions

Display This Question:

If What year are you? = HO-1

Q9 The following questions apply to the one-on-one chief intern meetings:

Display This Question:

If What year are you? = HO-1

Q10 The sessions have been helpful in the following areas:

|  | Strongly disagree (1) | Somewhat disagree (2) | Neither agree nor disagree (3) | Somewhat agree (4) | Strongly agree (5) |
| --- | --- | --- | --- | --- | --- |
| Normalization of intern experience (1) |  |  |  |  |  |
| Stress management (2) |  |  |  |  |  |
| Providing me with additional wellness resources (3) |  |  |  |  |  |
| Feeling connected to program leadership (4) |  |  |  |  |  |

Display This Question:

If What year are you? = HO-1

Q11 What about these sessions has been helpful?

________________________________________________________________

Display This Question:

If What year are you? = HO-1

Q12 How could the sessions be improved?

________________________________________________________________

Display This Question:

If What year are you? = HO-1

Q13 The different wellness topics that we discuss each month have given me additional tools to process intern year

- Strongly disagree (1)
- Somewhat disagree (2)
- Neither agree nor disagree (3)
- Somewhat agree (4)
- Strongly agree (5)

Display This Question:

If What year are you? = HO-1

Q14 Which discussion topics have been helpful? Which were not helpful? What other topics would you want to see discussed in these meetings?

________________________________________________________________

Display This Question:

If What year are you? = HO-1

Q15 Thank you for completing this survey.  We've really enjoyed meeting with all of you and getting to know you.  We look forward to continuing to meet with you and watch you progress through intern year.

End of Block: One On One Meeting Questions
